# A nationwide study of the incidence, prevalence and mortality of Parkinson’s disease in the Norwegian population

**DOI:** 10.1038/s41531-022-00280-4

**Published:** 2022-03-02

**Authors:** Brage Brakedal, Lilah Toker, Kristoffer Haugarvoll, Charalampos Tzoulis

**Affiliations:** 1 Neuro-SysMed, Department of Neurology, Haukeland University Hospital, 5021 Bergen, Norway; 2 Department of Clinical Medicine, University of Bergen, Pb 7804, 5020 Bergen, Norway

**Keywords:** Parkinson's disease, Epidemiology

## Abstract

Epidemiological studies of Parkinson’s disease (PD) show variable and partially conflicting findings with regard to incidence, prevalence, and mortality. These differences are commonly attributed to technical and methodological factors, including small sample sizes, differences in diagnostic practices, and population heterogeneity. We leveraged the Norwegian Prescription Database, a population-based registry of drug prescriptions dispensed from Norwegian pharmacies to assess the incidence, prevalence, and mortality of PD in Norway. The diagnosis of PD was defined based on the prescription of dopaminergic drugs for the indication of PD over a continuous time. During 2004–2017, 12,229 males and 9831 females met our definition for PD diagnosis. PD prevalence increased over the observation period, with larger changes observed in the older age groups. Incidence and prevalence of PD increased with age, peaking at 85 years. The male/female prevalence ratio was 1.5 across all ages, whereas the incidence ratio increased with age, from 1.4 in those 60 years, to 2.03 among those >90 years. While PD mortality was generally higher than that of the general population, mortality odds ratios decreased with age, approaching 1.0 among individuals >90 years old. When adjusted for the sex-specific mortality of the general population, the mortality among females with PD was equal to or higher than the mortality among males with PD. Our findings demonstrate that the epidemiological features of PD, including sex-differences, are age and time-period dependent and indicate that sex differences in PD mortality are unlikely to stem from disease-specific negative impact of survival in males.

## Introduction

Parkinson’s disease (PD) is the second most common neurodegenerative disorder, and the fastest-growing neurological disease in terms of prevalence, related disability, and mortality^1^. PD affects 1–2% of individuals above 65 and its prevalence is rapidly increasing as the population ages^2^. Since there are currently no neuroprotective therapies able to prevent or delay disease progression, PD is a major healthcare and societal challenge. Elucidating the epidemiology of PD is, therefore, essential for estimating its socioeconomic impact, enabling informed policy decision making, and shedding light on factors involved in the disease etiology.

Multiple studies have assessed descriptive epidemiology aspects of PD (e.g., incidence, prevalence, and mortality) in different populations world-wide. The incidence and prevalence of PD are consistently reported to increase with age^3–6^, though the exact numbers vary in different populations^1,3,4^ and conflicting observations have been made for individuals above the age of 80^6,7^. Furthermore, higher PD incidence and prevalence have been found in males compared to females^3,5,8^, although the male/female ratios vary greatly among studies^7,9,10^, with lower ratios reported from Asian populations^5,9^. Despite the importance of accurate mortality estimation for the evaluation of PD prognosis and health-service planning, current studies are largely inconclusive, reporting a wide range of mortality ratios and between-study heterogenity^11^. Moreover, the effect of sex on PD mortality has not been adequately explored. While several studies have shown higher mortality risk among males with PD compared to females, these have not taken into account the increased mortality risk for males in the general population^2,12^. Thus, it remains unknown whether PD-associated mortality differs between males and females.

Discrepancies between epidemiological studies are often attributed to small sample sizes, ascertainment bias, biological variation (e.g., population heterogeneity), and methodological differences such as practices and/or changes in diagnostic criteria over time^6,7,9,13^. To minimize technical variation and improve the quality and reliability of the observations, there is a need to study large, unselected, and genetically and environmentally homogeneous populations. Digital health records, health databases, and drug registries offer an easy and reliable way to continuously monitor disease epidemiology on a population-wide level^14–18^. In the current study, we utilized the Norwegian Drug Prescription Database (NorPD), to assess the incidence, prevalence and mortality of PD in the entire Norwegian population between 2004 and 2017. Our data are representative of the entire Norwegian population, which is relatively homogeneous in terms of both genetic background and environmental exposures.

## Results

Between the years 2005 and 2017, 15,732 individuals met our criteria for PD (6841 females and 8891 males). Of these, 5979 (2531 females and 3448 males) died during the observation period. The median age of onset was 73 for females, and 72 for males. Among the PD individuals who met our criteria, 96% received more than 6 dopaminergic prescriptions, and 68% received more than 20 dopaminergic prescriptions. Additional 6328 individuals meeting our criteria for PD were identified when 2004 data were included in the analyses. The vast majority of these were most likely cases diagnosed prior to 2004. In line with this assumption, 82% (5170) of these individuals died during the observation period (compared to 38% of the individuals with first registered drug after 2004).

### Incidence and prevalence of PD in the Norwegian population between 2005 and 2016

**Fig. 1:**
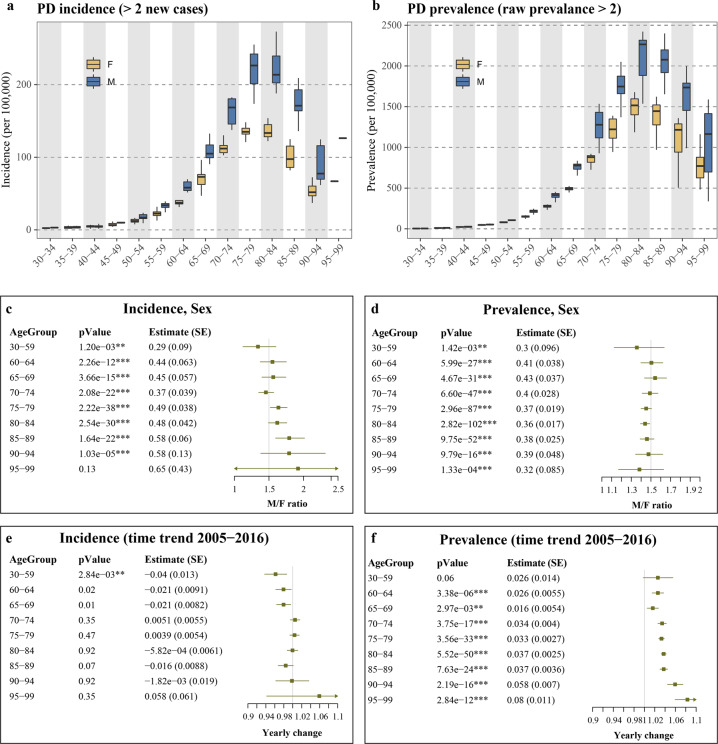
Incidence and prevalence of PD in the Norwegian population during 2005–2016. **a**, **b** Incidence and prevalence were first calculated for each observation year by dividing the number of new or total PD cases by the corresponding age- and sex-matched population size in the same year. For presentation purposes, the measures were summarized by age groups. Data points for which the number of new cases/raw prevalence were <2, were excluded. X-axis indicates the age group of the individuals. **c**–**f** The impact of sex (**c**, **d**) and time (**e**, **f**) were assessed individually for each age group, using Poisson regression. The *p*-values and the estimates for each covariate are shown in tables (for time trend, the estimate represents the natural log of the estimated change in each sequential year). SE, standard error. M/F ratio and Yearly change were derived by taking the exponential of the estimate for each covariate (sex or year). Lines represent the 95% confidence interval.

The crude incidence for PD between 2005 and 2016 was on average 23.1 for females and 29.6 for males, per 100,000 person-years. The prevalence for PD in the population was on average 0.2% of the females and 0.23% of the males in the general population, and 0.98% of the females and 1.35% of the males for the population >65 years. For both sexes, the age-specific incidence and prevalence increased with age, peaking at the 75–85 age group (Table 1, Fig. 1a, b, Supplementary Fig. 1). However, while the male/female PD prevalence ratio remained ~1.5 across all age groups (Fig. 1d), the male/female incidence ratio changed with age, increasing by 1.2% for every year of life (ANOVA comparing the models with or without Age × Sex interaction, *p* < 9.9 × 10^−8^; negative binomial regression adjusted for time-trend, age and sex: *β*_Age × Sex_ = 0.012, *p* = 5.76 × 10^−10^, Fig. 1c, Supplementary Fig. 2). Substantial variation in both incidence and prevalence was observed over the 2005–2016 observation period, for which the measures were calculated (“Methods” section and Supplementary Table 1). There was no general time-trend in the incidence of PD during the observation period, though a significant decrease (*p* = 0.0028) was observed among the 30–59 age group (Fig. 1e). In contrast, PD prevalence significantly increased during the observation period in all age groups, with the exception of the 30–59 group, for which only a trend for increased prevalence was observed (*p* = 0.06). Interestingly, the yearly rise in PD prevalence increased with age, with the biggest differences observed for older populations (Fig. 1f).

**Table 1:** Age- and sex-adjusted PD incidence, prevalence, and mortality.

Sex	Age group	Incidence	Prevalence	Mortality
Females	30–34	0 (0–2.5)	1.8 (0–4.7)	36 (29–43)
	35–39	2 (0–5.8)	10.8 (4.2–16.2)	51 (41–69)
	40–44	5.2 (1.6–6.9)	23.3 (10.5–29.7)	80 (66–104)
	45–49	6.5 (4.2–14)	48 (19.9–53.9)	139 (106–175)
	50–54	12.3 (7.7–22.3)	80 (54.7–91.5)	241 (197–271)
	55–59	22.4 (12.9–35.6)	151 (114–167)	357 (328–409)
	60–64	37.4 (31.4–57.3)	280 (191–300)	595 (526–685)
	65–69	73 (47.1–96.3)	496 (420–524)	961 (818–1002)
	70–74	112 (102.8–134.3)	880 (654–921)	1564 (1394–1612)
	75–79	135.1 (118.1–148.2)	1222 (945–1389)	2709 (2576–2969)
	80–84	133.4 (122.2–153.9)	1516 (1021–1678)	5284 (4797–5692)
	85–89	97.5 (82–124.9)	1446 (971–1623)	10,273 (9511–11,450)
	90–94	51.8 (22–72.4)	1216 (506–1359)	20,311 (18,778–21,611)
	95–99	15.3 (0–66.9)	772 (484–1162)	35,003 (32,618–36,415)
Males	30–34	0.3 (0–3.5)	2.1 (0–5.9)	80 (71–97)
	35–39	2.2 (1.1–5.5)	12 (3.9–17.8)	99 (71–123)
	40–44	4.5 (1.6–8.3)	25.1 (15–36)	134 (109–153)
	45–49	9.9 (7.1–12.9)	52.3 (43.9–65.6)	214 (174–247)
	50–54	16.9 (9.2–23.5)	106 (84–114)	351 (301–392)
	55–59	33.8 (24.3–39.2)	221 (175–247)	574 (471–629)
	60–64	58.2 (51.6–84)	415 (326–454)	938 (801–1104)
	65–69	105 (90.5–132.6)	777 (617–836)	1627 (1315–1736)
	70–74	168.6 (137.6–182.5)	1278 (928–1535)	2556 (2271–2893)
	75–79	226.4 (173.6–255.1)	1748 (1371–2050)	4479 (3691–5147)
	80–84	213.7 (187.9–273)	2265 (1537–2422)	8250 (6982–9172)
	85–89	171 (136–238.3)	2077 (1485–2399)	14,702 (13,348–17,000)
	90–94	76 (43.8–124.6)	1735 (990–1998)	26,709 (24,420–28,602)
	95–99	12.8 (0–266.7)	1165 (338–1588)	40,810 (38,997–43,731)

Values were first calculated per 100,000 individuals, during each observation year and for each age separately, and then aggregated into age groups. Shown are the median and the range of each of the yearly values during the observation period. The raw data are provided as Supplementary Table 1.

### PD mortality in the Norwegian population between 2005 and 2016

**Fig. 2:**
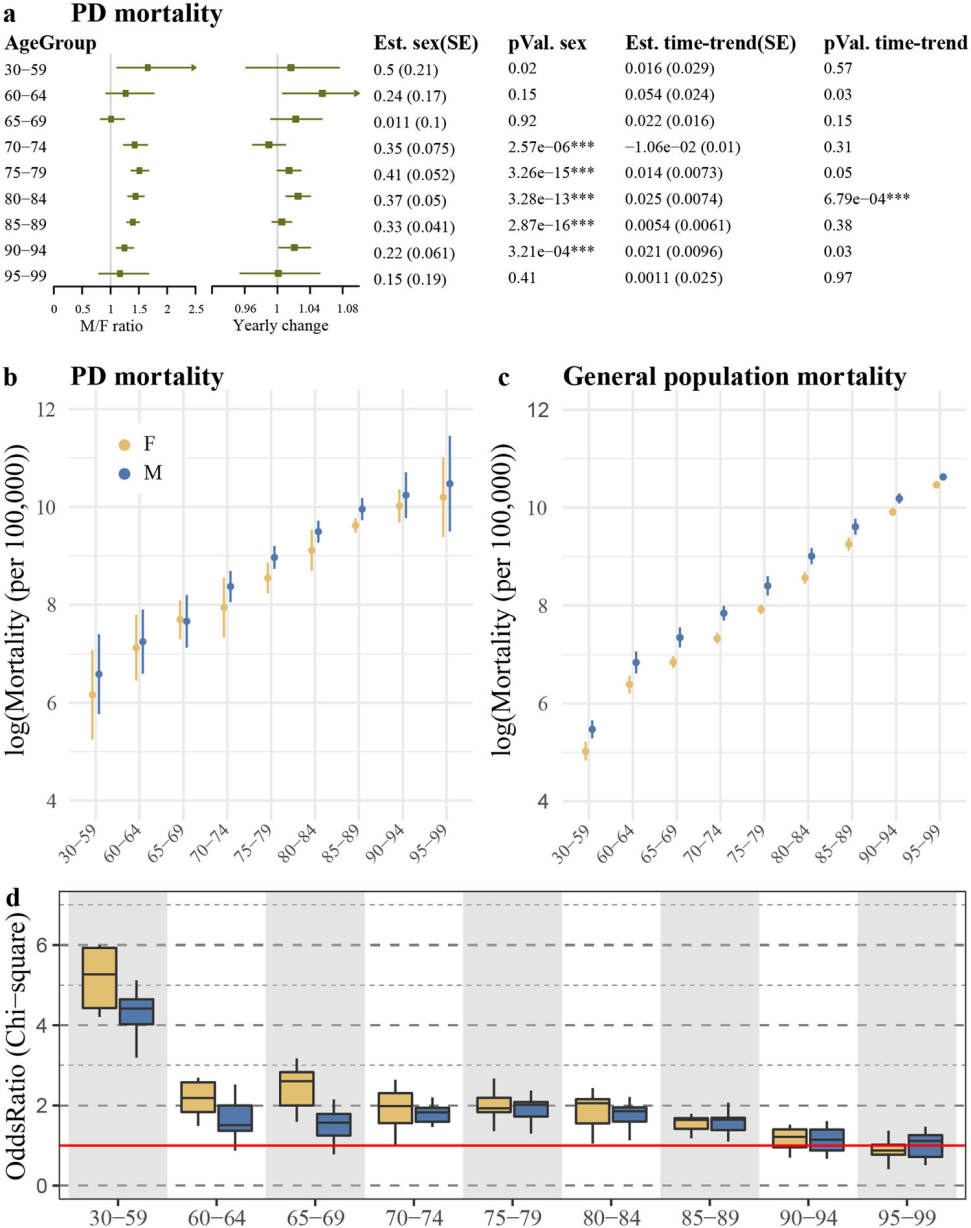
Mortality of PD in the Norwegian population during 2005–2016. **a** The impact of the time period and sex on PD mortality were assessed individually for each age group, using Poisson regression. The *p*-values and the estimates for each covariate are shown in the tables. SE, standard error. M/F ratio and yearly change were derived by taking the exponential of the estimate for each covariate (sex or year). Lines represent the 95% confidence interval. **b**, **c** Natural log of mortality per 100,000 person-years in PD (**b**) and general population (**c**). The points indicate the mean mortality during 2005–2016, and the line indicates two standard deviations. **d** Death odds ratio of individuals with PD compared to general population were calculated for each year, separately for each age group, for females (yellow) and males (blue). Boxplots show the estimated odds ratios of the observation years. Red line indicates odds ratio of 1. The estimated odds ratio and the 95% confidence intervals for each observation year are shown in Supplementary Fig. 3.

**Fig. 3:**
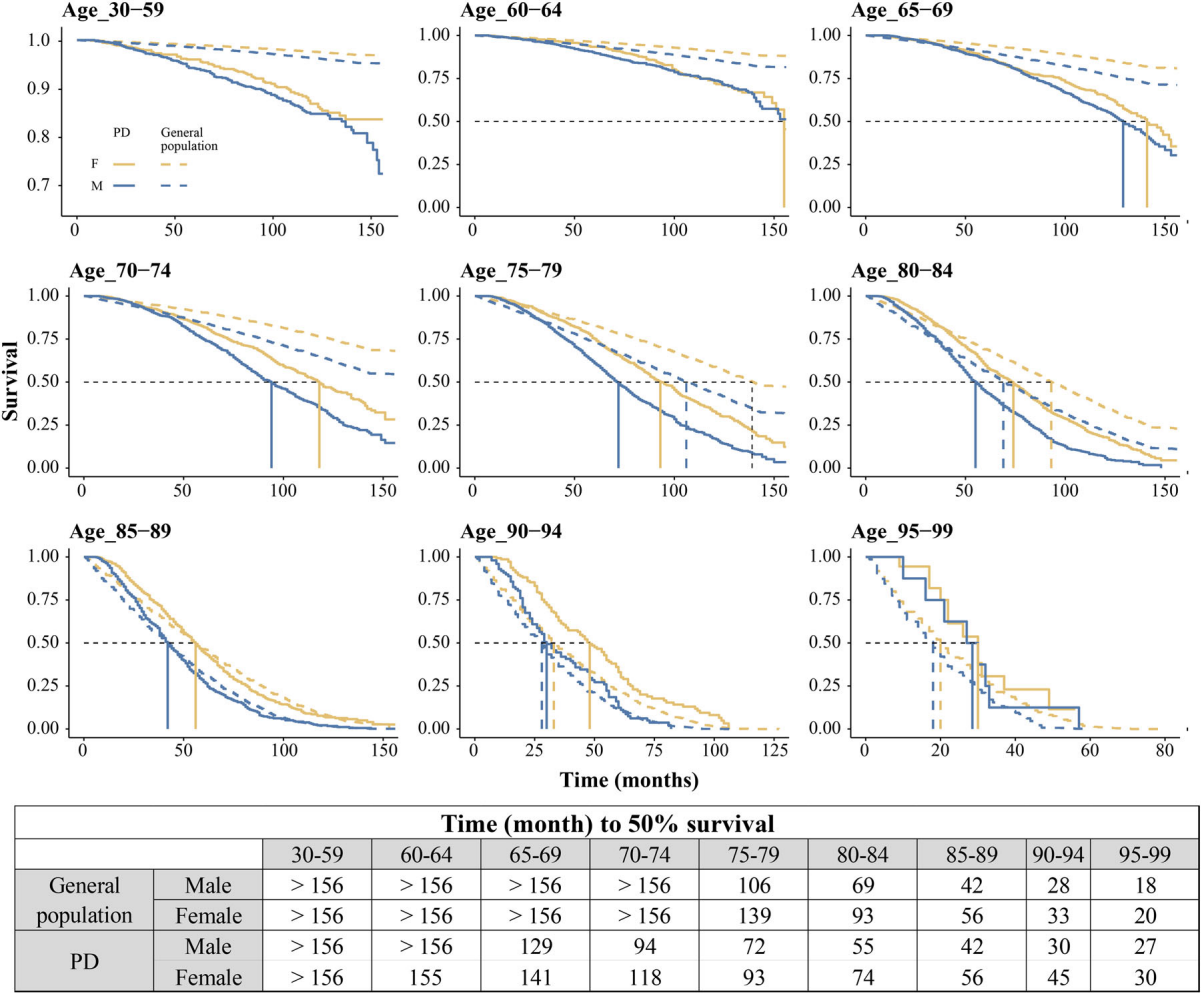
Survival analysis showing sex differences. Kaplan–Meier survival curves for males (blue) and females (yellow) with PD (solid line) and general population (dashed line) in Norway 2005–2017. In both sexes, the differences in survival between individuals with PD and general population is only apparent among individuals with diagnosis age <85. Black dashed line represents 50% survival.

PD mortality was generally higher in males than females (Fig. 2a, b, Table 1), though the sex differences were more pronounced among the older age groups and were consistently lower than the sex differences observed in general population (Fig. 2b, c). In contrast to the observed sex differences in PD mortality, the death odds ratios (PD vs. general population) were similar between the two sexes among individuals older than 70 and higher in females compared to males among individuals younger than 70 years (Fig. 2d, Supplementary Fig. 3). In line with this observation, Kaplan–Meier survival analysis indicated no major differences in the survival ratio (PD vs. general population) between the two sexes (Fig. 3). Finally, in both sexes, the survival among individuals who were diagnosed with PD at 85 or older was similar to general population (Fig. 3).

## Discussion

We assessed epidemiological features of PD in the entire Norwegian population, over a 12-year period (2005–2016). We show that PD epidemiology is highly dynamic, and changes dramatically as a function of both age and time. The finding that PD prevalence increases with time in all age groups indicate that the observed world-wide increase in PD prevalence cannot be exclusively attributed to the aging of the population.

In line with previous reports, our findings indicate that, in both sexes, the incidence and the prevalence of PD increase with age, peaking around 85 years. The observed decline after 85 can at least partially be explained by technical reasons. Most importantly, NorPD lacks data on prescriptions dispensed to individuals residing in nursing homes, who typically are in the higher age groups. Thus, older individuals are more likely to be missed by our study design, which would explain the slightly lower incidence rates among individuals above 80 in our study, compared to other registry studies^19–21^. Second, the dramatic increase in incidence and prevalence of dementia during the last decade of life^22–24^, impacts the diagnosis of PD among elderly, since the existence of dementia prior to the onset of motor symptoms excludes the diagnosis of PD^5,25^. Finally, our exclusion of individuals with treatment period <6 months, while increasing our diagnostic specificity, removes individuals who died shortly after the beginning of the treatment. This scenario is of course much more likely to take place among elderly individuals.

Interestingly, while our findings are in line with previous studies indicating a higher PD incidence among males^3,5,8^, they also suggest that the male/female ratio is not constant but increases with age, ranging from 1.36 in the age group 30–59, to 1.79 in the age group 90–94 (Fig. 1c). Similar age-dependent increase in the male/female incidence was also demonstrated in Moisan et al.^26^, based on data collected in France during a single year (2010) and further supported by the authors using meta-analysis^26^. In contrast to Moisan et al., however, we did not observe an age-dependent change in the male/female ratio of the PD prevalence, which in our study remained ~1.5 across all age groups.

As expected, mortality among individuals with PD was higher than in the general population with the exception of individuals >85 years old, whose mortality was comparable to that of the general population, both in terms of crude mortality (Fig. 2b–d) and survival time (Fig. 3). The most likely explanation for this observation is the competing risk of death from age-related comorbidities. In line with previous reports^27,28^, PD mortality was higher in males compared to females. However, the 50% survival ratio between PD and the general population was similar among males and females, corroborating two previous studies^29,30^. Furthermore, the death odds ratio among PD individuals younger than 70 was higher in females than in males, when compared to the general population. A similar observation was recently reported in the Australian population^30^. This emphasizes the importance of taking into account the baseline mortality in the relevant subgroups of the general population when estimating the impact of different factors on disease mortality.

In both sexes and across all age groups, the prevalence of PD increased during the observation years, without concomitant changes in either incidence or mortality. This apparent paradox can be explained by an increase in the incidence and/or a decrease in mortality of PD occurring prior to our observation period (i.e., before 2004). Similar time-trends during the period 2005–2011, were reported in the Taiwanese population^20^. Increase in the prevalence of PD between 1990 and 2016 was also reported from the Global Burden of Disease study^1^, and was predicted by Wanneveich et al.^31^ to occur even under the constrain of constant incidence. In line with the observation made by Liu et al.^20^, the increase in prevalence of PD over the observation period in our study was more prominent among older age groups (Fig. 1d). This age dependency is consistent with a scenario in which an increase in PD incidence and/or a decrease in mortality took place prior to the observation period, since older age groups include a higher proportion of individuals diagnosed prior to 2004. Supplementary Fig. 4 shows simulated data demonstrating this potential scenario. In line with this hypothetical scenario, an increase in the incidence of PD prior to 2005 was reported based on three decades of observations in the North American population^32^. While the cause for this increase remains unknown, it can be potentially attributed to the world-wide decline in tobacco smoking^33,34^, since smoking has been shown to decrease the risk of PD^35,36^. Interestingly, starting from the late 90 s’, Norway saw a gradual increase in the use of moist smokeless tobacco (snus)^37^, which has also been suggested to decrease the risk of PD^38–41^, although this hypothesis has yet to be confirmed. While speculative, it is thus possible that the observed increase in PD prevalence, without concordant change in incidence during our observation period, was at least partly mediated by gradual decrease in smoking, more recently substituted by snus usage. Another potential explanation for a tentative increase in the incidence of PD prior to 2004 is higher diagnostic sensitivity and specificity. With the advent of advanced imaging methods, such as DaTscan^42^ and, in part, cardiac 123I-MIBG scintigraphy^43^, being increasingly utilized in Norway since the late 90 s, it is plausible that both diagnostic sensitivity and, partly, accuracy improved in that period. Finally, a potential decrease in PD-related mortality prior to 2004 may also contribute to the observed increase in prevalence during our observation period. While no data on PD mortality is available to us before 2004, advanced PD treatment, in the form of deep brain stimulation (DBS), was introduced to Norway in 1999^44^. DBS has been shown to confer a significant survival advantage, even in patients with advanced/severe disease^45^. Thus, a DBS-mediated decrease in mortality for individuals with PD in the period 1999–2004 in our population is plausible.

Changes in the incidence of PD point to temporal appearance or disappearance of risk factors simultaneously affecting large proportion of the population. However, since risk factors may act decades before the onset of the disease, they may be difficult to identify. For example, while the 1918 Spanish flu pandemic was linked to post-encephalitis parkinsonism^46,47^, whether or not this pandemic resulted in increased incidence of PD decades later remains unknown. Similarly, concern has been raised that the current COVID-19 pandemic could lead to a world-wide increase in idiopathic PD^47,48^. Such an effect may only become apparent several years, or even decades, into the future.

A major advantage of our study is that it is population-based and represents time and age-trends in a relatively homogeneous population, in terms of both genetic background and environmental exposures. Namely, the variation in our data is more likely to be due to time-dependent environmental changes, rather than ethnical or geographical variation. It is also unlikely that differences in diagnostic or treatment practices over time have a substantial impact on our data since no major changes in diagnostic or primary drug treatment practices were introduced in Norway during the observation period.

Our study has several limitations. Misclassification of PD in our data is possible, as the classification was done retrospectively. The main source of misclassification would be patients with secondary parkinsonism or Parkinson-plus syndromes, originally misdiagnosed with PD and treated with dopaminergic drugs. We note, however, that these individuals generally have a poor and/or short-lived symptomatic response^49^. Thus, our requirement of at least three consecutive prescriptions with a minimal interval of 6 months should minimize these misclassifications, since nationally applied treatment algorithms in Norway dictate that dopaminergic therapy is discontinued if ineffective. Important exceptions to this rule are the parkinsonian types of multiple system atrophy (MSA-P) and progressive supranuclear palsy (PSP-P). These commonly show a positive dopaminergic response over longer periods of time and are clinical more similar to PD and, therefore, more likely to be misclassified as PD in our study. Even so, the collective incidence of Parkinson-plus syndromes and other parkinsonisms is substantially lower than that of PD^50,51^. Thus, even if some patients with these diseases were included in our study, this would be highly unlikely to have a major impact on our results.

Another potential source of bias is missing data from untreated PD patients. However, untreated PD patients are extremely rare in Norway, where welfare-based systematic follow-up and treatment is offered to all patients with PD, nationwide, and all treatment is fully reimbursed. In fact, the only two scenarios where a PD patient would not receive dopaminergic therapy in Norway, would be either the lack of response, which should prompt a different diagnosis, or severe side-effects even at small doses, which would concern mostly institutionalized patients with very advanced disease, who are irrespective not included in our analyses.

The accuracy of our classification method is supported by the fact that the observed age-specific incidence rates are very similar to those previously reported in other populations^6,26^. Our age-specific incidence rates are highly similar to those reported by the highest quality studies, employing multiple follow-up visits, prospective design, and multiple sources of ascertainment, included in the meta-analysis by Hirsch et al.^6^. Furthermore, the age-adjusted prevalence of PD in our data is highly similar to that reported in previous studies from other populations^4,9^, in particular to the pooled estimate of the highest quality studies as reported in the meta-analysis by Pringsheim et al.^9^.

To summarize, we report that sex, age, and the time period have a major impact on the incidence, prevalence, and mortality of PD in the Norwegian population. While our observed age-adjusted incidence, prevalence, and mortality numbers are similar to other epidemiological studies of PD, we show that these measures can change dramatically with time and/or age. Our study indicates that the previously reported increase in prevalence of PD cannot be explained merely by the global aging of the population, since the increase takes place among all age groups. Furthermore, we demonstrate that the mortality among males with PD is not greater than that of females with PD when accounting for the sex-specific mortality of the general population. Finally, the high dependency of epidemiological measures on the time period, age, and sex of the individual indicates that meta-analyses combining studies from different age groups or time periods might be misleading, as they would average and potentially cancel-out important biological effects. It is therefore essential that future epidemiological research on PD accounts for these factors through data stratification.

## Methods

### Data acquirement

This study based on NorPD, a population-based registry of all drug prescriptions dispensed from Norwegian pharmacies initiated on 01/01/2004. NorPD comprises a complete record of every dispensing of prescribed medication from pharmacies for the entire Norwegian population (4.6–5.3 million during the observation period)^52^, a relatively homogeneous white Caucasian population of north Germanic descent. We obtained from NorPD all anti-Parkinson drugs (ATC Code N04*) dispensed between 01/01/2004 and 31/12/2017. The clinical indications for which the prescription was given are registered in the form of either a diagnosis code from the International Classification of Diseases, 10th revision (ICD-10), and/or the International Classification of Primary Care, 2nd edition (ICPC-2), or a disease or disease-group specific reimbursement code. Anti-PD medication is reimbursed in Norway and strictly prescription-controlled. A complete record of deaths is included in the NorPD. PD incidence was defined based on the use of levodopa (ATC Code: N04BA02, N04BA03), monoamine oxidase B inhibitors (ATC Code: N04BD01, N04BD02, N04BD03), or dopamine agonist (ATC Code: N04BC04, N04BC05, N04BC09), either alone or in combination, prescribed specifically for the indication of PD, and dispensed at least three consecutive times and at least 30 days apart. The time from the first to the last dopaminergic prescription had to be at least 180 days. All dopaminergic drug prescriptions given for indications other that PD (e.g., restless legs syndrome) were excluded. Further exclusion criterion was the combination of the following three conditions: treatment duration <1 year and off-treatment period >2 years (the time difference between the date of the last dispensed medication and the date of death/end of record period) and age of onset >80. The age conditioning in the exclusion criterion is because among older individuals long off-treatment period captured through NorPD is likely to be a result of administration into nursing home. In addition, we excluded individuals with age of diagnosis <30 or >100. For each individual, time of PD diagnosis was set to the first dispensed dopaminergic medication if they fulfilled criteria for inclusion. Prevalence of PD to a subject was defined during the time from their incidence of PD until one of the endpoints; death or end of observation (31.12.2017).

Population data was downloaded from Statistisk sentralbyrå (Statistics Norway), the official site of population statistics in Norway (https://www.ssb.no/en/statbank).

### Statistical analyses

Incidence, prevalence, and mortality (presented per 100,000 person-years) were first calculated separately for each age, in each observation year, and then summarized in age groups of five, by taking the mean of the values for each age group. The calculations for each year (*y*), age (*a*), and sex (*s*) were done as following: Incidence = 10^5^*New_cases_*yas*_/Total_population_*yas*_; Prevalence = 10^5^*All_cases_*yas*_/Total_population_*yas*_; Mortality = 10^5^*Deaths_*yas*_/Prevalence_*yas*_. Since even among the older age groups PD prevalence is <3%, we did not retract raw PD prevalence from the total population for incidence calculation, since it would have a negligible impact on our calculations. No measures were calculated for 2004 and 2017, because the minimum requirement of three prescriptions could not be adequately assessed for these years, and it was not possible to differentiate between new and existing cases in 2004. Since the yearly number of individuals with PD younger than 60 years in each age group was small (<4 individuals), for some of the analyses, these individuals were grouped into a single age group: 30–59.

The effects of sex and time period on the incidence, prevalence, and mortality were assessed for each age group separately through Poisson regression using the “glm” function from the “stats” basic R package, by setting the family variable to “poisson”. Alternatively, when over-dispersion was observed (dispersion >1.5), the estimates were assessed through negative binomial distribution using the “glm.nb” function from the R package “MASS” v7.3-53. Death odds ratios were calculated for each year in each age group (separately for males and females) through Chi-square analysis using the “oddsratio” function from the R package “epitools” v0.3.1. Kaplan–Meier plots were created using “survfit” and “ggsurvplot” functions from the “survival” v3.2-7 and “suvminer” v0.4.8R packages. For the purpose of the survival analyses, the survival in the general population was simulated using the general population size in 2005 as baseline and propagating individual death dates using the annual age and sex specific death data downloaded from Statistics Norway (https://www.ssb.no/en/statbank). While this simulation does not take into account migration flows, this is unlikely to introduce bias in our analysis due to the small crude rate of migration in Norway https://www.ssb.no/en/statbank/table/09203/tableViewLayout1/.

All analyses were performed using R software version 4.0.3. The data used for this study and all scripts required to reproduce the analyses and the figures are accessible through the repository https://github.com/ltoker/PDepidemiology. Detailed information regarding all versions used for the analysis is provided in “SessionInfo.Rds” file available through the github repository.

### Reporting summary

Further information on research design is available in the Nature Research Reporting Summary linked to this article.

## Supplementary information

Supplementary Information

Reporting Summary

## Data Availability

The data that supports these findings is available through the repository https://github.com/ltoker/PDepidemiology.

## Code availability

All scripts required to reproduce the analyses and the figures are accessible through the repository https://github.com/ltoker/PDepidemiology. Detailed information regarding all versions used for the analysis is provided in “SessionInfo.Rds” file available through the github repository.

## References

[1] Dorsey ER (2018). Global, regional, and national burden of Parkinson’s disease, 1990–2016: a systematic analysis for the Global Burden of Disease Study 2016. Lancet Neurol..

[2] de Rijk MC (2000). Prevalence of Parkinson’s disease in Europe: a collaborative study of population-based cohorts. Neurologic Diseases in the Elderly Research Group. Neurology.

[3] Abbas MM, Xu Z, Tan LC (2018). Epidemiology of Parkinson’s disease—East versus West. Mov. Disord. Clin. Pract..

[4] Marras C (2018). Prevalence of Parkinson’s disease across North America. NPJ Parkinson’s Dis..

[5] Ascherio A, Schwarzschild MA (2016). The epidemiology of Parkinson’s disease: risk factors and prevention. Lancet Neurol..

[6] Hirsch L, Jette N, Frolkis A, Steeves T, Pringsheim T (2016). The incidence of Parkinson’s disease: a systematic review and meta-analysis. Neuroepidemiology.

[7] Bower JH, Maraganore DM, McDonnell SK, Rocca WA (2000). Influence of strict, intermediate, and broad diagnostic criteria on the age- and sex-specific incidence of Parkinson’s disease. Mov. Disord..

[8] Wirdefeldt K, Adami HO, Cole P, Trichopoulos D, Mandel J (2011). Epidemiology and etiology of Parkinson’s disease: a review of the evidence. Eur. J. Epidemiol..

[9] Pringsheim T, Jette N, Frolkis A, Steeves TDL (2014). The prevalence of Parkinson’s disease: a systematic review and meta-analysis. Mov. Disord..

[10] Gillies GE, Pienaar IS, Vohra S, Qamhawi Z (2014). Sex differences in Parkinson’s disease. Front Neuroendocrinol..

[11] Macleod AD, Taylor KS, Counsell CE (2014). Mortality in Parkinson’s disease: a systematic review and meta-analysis. Mov. Disord..

[12] Xu J, Gong DD, Man CF, Fan Y (2014). Parkinson’s disease and risk of mortality: meta-analysis and systematic review. Acta Neurologica Scandinavica.

[13] de Rijk MC (1997). A population perspective on diagnostic criteria for Parkinson’s disease. Neurology.

[14] Pasternak B (2012). Use of calcium channel blockers and Parkinson’s disease. Am. J. Epidemiol..

[15] Manthripragada AD (2011). Non-steroidal anti-inflammatory drug use and the risk of Parkinson’s disease. Neuroepidemiology.

[16] Brauer R (2020). Diabetes medications and risk of Parkinson’s disease: a cohort study of patients with diabetes. Brain.

[17] Brakedal B (2017). Glitazone use associated with reduced risk of Parkinson’s disease. Mov. Disord..

[18] Liu C-C, Li C-Y, Lee P-C, Sun Y (2016). Variations in incidence and prevalence of Parkinson’s disease in Taiwan: a population-based nationwide study. Parkinson’s Dis.

[19] Blin P (2015). Parkinson’s disease incidence and prevalence assessment in France using the national healthcare insurance database. Eur. J. Neurol..

[20] Liu W-M (2016). Time trends in the prevalence and incidence of Parkinson’s disease in Taiwan: a nationwide, population-based study. J. Formos. Med. Assoc..

[21] Jones CA, Martin WW, Wieler M, King-Jesso P, Voaklander DC (2012). Incidence and mortality of Parkinson’s disease in older Canadians. Parkinsonism Relat. Disord..

[22] Fratiglioni L, De Ronchi D, Agüero-Torres H (1999). Worldwide prevalence and incidence of dementia. Drugs Aging.

[23] Ferri CP (2005). Global prevalence of dementia: a Delphi consensus study. Lancet.

[24] Matthews FE (2016). A two decade dementia incidence comparison from the Cognitive Function and Ageing Studies I and II. Nat. Commun..

[25] Postuma RB (2015). MDS clinical diagnostic criteria for Parkinson’s disease. Mov. Disord..

[26] Moisan F (2016). Parkinson disease male-to-female ratios increase with age: French nationwide study and meta-analysis. J. Neurol. Neurosurg. Psychiatry.

[27] Berger K (2000). Prognosis with Parkinson’s disease in europe: a collaborative study of population-based cohorts. Neurologic Diseases in the Elderly Research Group. Neurology.

[28] Diem-Zangerl A (2009). Mortality in Parkinson’s disease: a 20-year follow-up study. Mov. Disord..

[29] Herlofson K, Lie SA, Arsland D, Larsen JP (2004). Mortality and Parkinson disease: a community based study. Neurology.

[30] Poortvliet PC, Gluch A, Silburn PA, Mellick GD (2021). The Queensland Parkinson’s Project: an overview of 20 years of mortality from Parkinson’s disease. J. Mov. Disord..

[31] Wanneveich M, Moisan F, Jacqmin‐Gadda H, Elbaz A, Joly P (2018). Projections of prevalence, lifetime risk, and life expectancy of Parkinson’s disease (2010–2030) in France. Mov. Disord..

[32] Savica R, Grossardt BR, Bower JH, Ahlskog JE, Rocca WA (2016). Time trends in the incidence of Parkinson’s disease: a 30-year study. JAMA Neurol..

[33] Lund KE, Lund M, Bryhni A (2009). Tobacco consumption among men and women 1927–2007. Tidsskr Nor Laegeforen.

[34] National Center for Chronic Disease Prevention and Health Promotion (US) Office on Smoking and Health. *Fifty Years of Change 1964–2014*. *The Health Consequences of Smoking—50 Years of Progress: A Report of the Surgeon General* (Centers for Disease Control and Prevention (US), 2014). 24455788

[35] Noyce AJ (2012). Meta-analysis of early nonmotor features and risk factors for Parkinson disease. Ann. Neurol..

[36] Mappin-Kasirer B (2020). Tobacco smoking and the risk of Parkinson disease: a 65-year follow-up of 30,000 male British doctors. Neurology.

[37] Lund KE, McNeill A (2013). Patterns of dual use of snus and cigarettes in a mature snus market. Nicotine Tob. Res..

[38] Yang F (2017). Moist smokeless tobacco (Snus) use and risk of Parkinson’s disease. Int J. Epidemiol..

[39] Benedetti MD (2000). Smoking, alcohol, and coffee consumption preceding Parkinson’s disease: a case-control study. Neurology.

[40] Brolin K (2022). Insights on genetic and environmental factors in Parkinson’s disease from a regional Swedish case-control cohort. J. Parkinsons Dis.

[41] Liu Z, Roosaar A, Axéll T, Ye W (2017). Tobacco use, oral health, and risk of Parkinson’s disease. Am. J. Epidemiol..

[42] Seifert KD, Wiener JI (2013). The impact of DaTscan on the diagnosis and management of movement disorders: a retrospective study. Am. J. Neurodegener. Dis..

[43] Skowronek C, Zange L, Lipp A (2019). Cardiac 123I-MIBG scintigraphy in neurodegenerative Parkinson syndromes: performance and pitfalls in clinical practice. Front Neurol..

[44] Toft M (2008). Behandling av bevegelsesforstyrrelser med dyp hjernestimulering. Tidsskrift for Den norske legeforening.

[45] Ngoga D (2014). Deep brain stimulation improves survival in severe Parkinson’s disease. J. Neurol. Neurosurg. Psychiatry.

[46] Henry J, Smeyne RJ, Jang H, Miller B, Okun MS (2010). Parkinsonism and neurological manifestations of influenza throughout the 20th and 21st centuries. Parkinsonism Relat. Disord..

[47] Monje MHG, Martínez‐Fernández R (2020). Severe acute respiratory syndrome coronavirus 2 infection and parkinsonism: is there evidence for concern?. Mov. Disord..

[48] Sulzer D (2020). COVID-19 and possible links with Parkinson’s disease and parkinsonism: from bench to bedside. NPJ Parkinsons Dis.

[49] Constantinescu R, Richard I, Kurlan R (2007). Levodopa responsiveness in disorders with parkinsonism: a review of the literature. Mov. Disord..

[50] Winter Y (2010). Incidence of Parkinson’s disease and atypical parkinsonism: Russian population-based study. Mov. Disord..

[51] Savica R, Grossardt BR, Bower JH, Ahlskog JE, Rocca WA (2013). Incidence and pathology of synucleinopathies and tauopathies related to parkinsonism. JAMA Neurol..

[52] National population projections. *SSB* https://www.ssb.no/en/befolkning/befolkningsframskrivinger/statistikk/nasjonale-befolkningsframskrivinger (2020).

