# Supplementary material for: A nationwide study of the incidence, prevalence and mortality of Parkinson’s disease in the Norwegian population
Source: NPJ Parkinsons Dis. 2022 Mar 2;8:19. doi: 10.1038/s41531-022-00280-4 (PMC8891365; doi:10.1038/s41531-022-00280-4)
Supplement: Supplementary file 1 — Supplementary Information [file 41531_2022_280_MOESM1_ESM.pdf]

Supplementary information

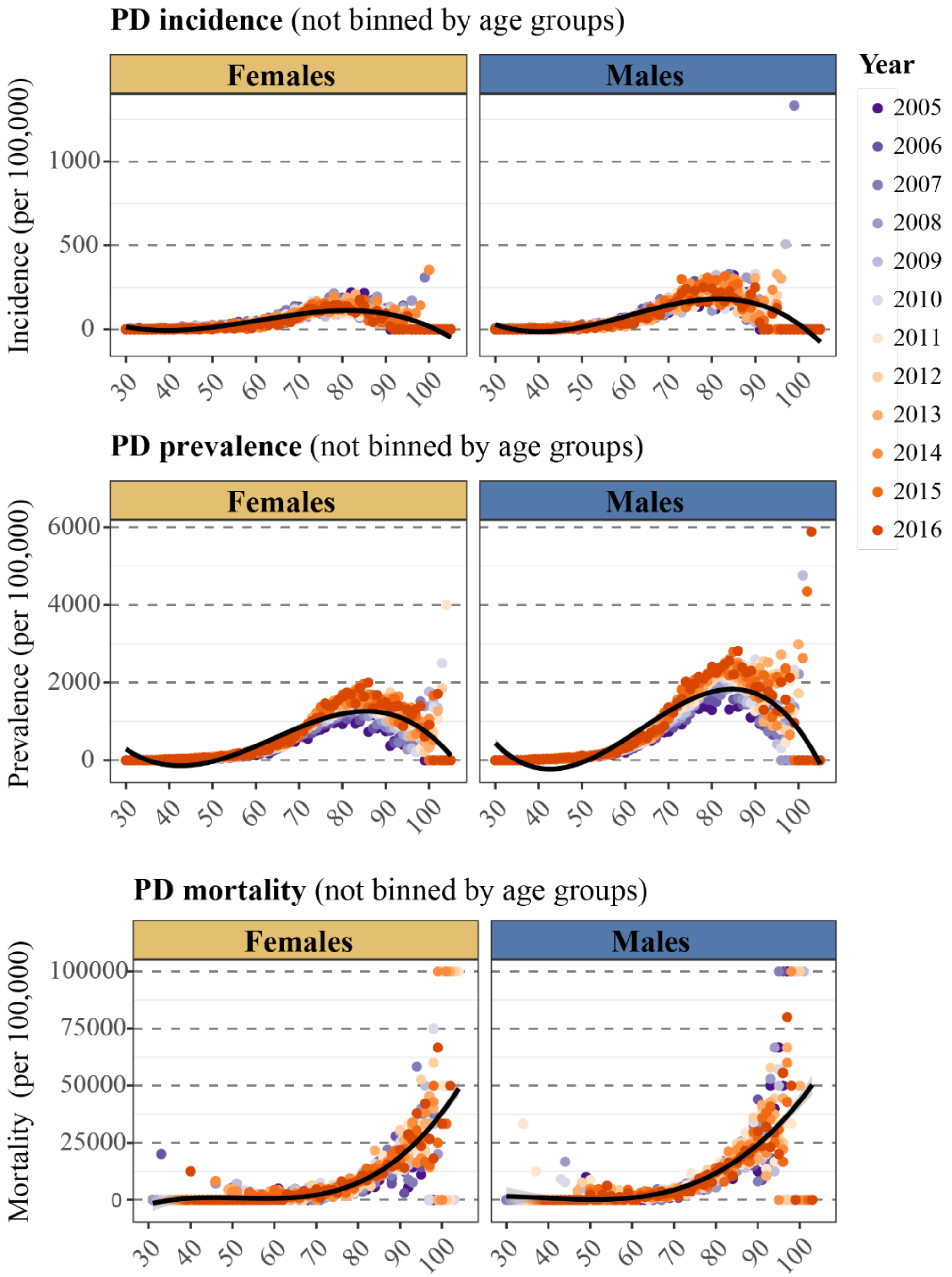

Supplementary Figure 1. Incidence, prevalence and mortality of PD in the Norwegian population without binning by age groups.

Each point represents the measured value (indicated on the y axis) in a specific year, for specific age (x axis). The measures are calculated per 100,000 person years.

All three measures are increasing with age. In both males and females, PD incidence and prevalence peak ~85. All three measures exhibit high variability throughout the 12 observation years, with prevalence (middle panel) exhibiting profound and consistent increase during the observation years.

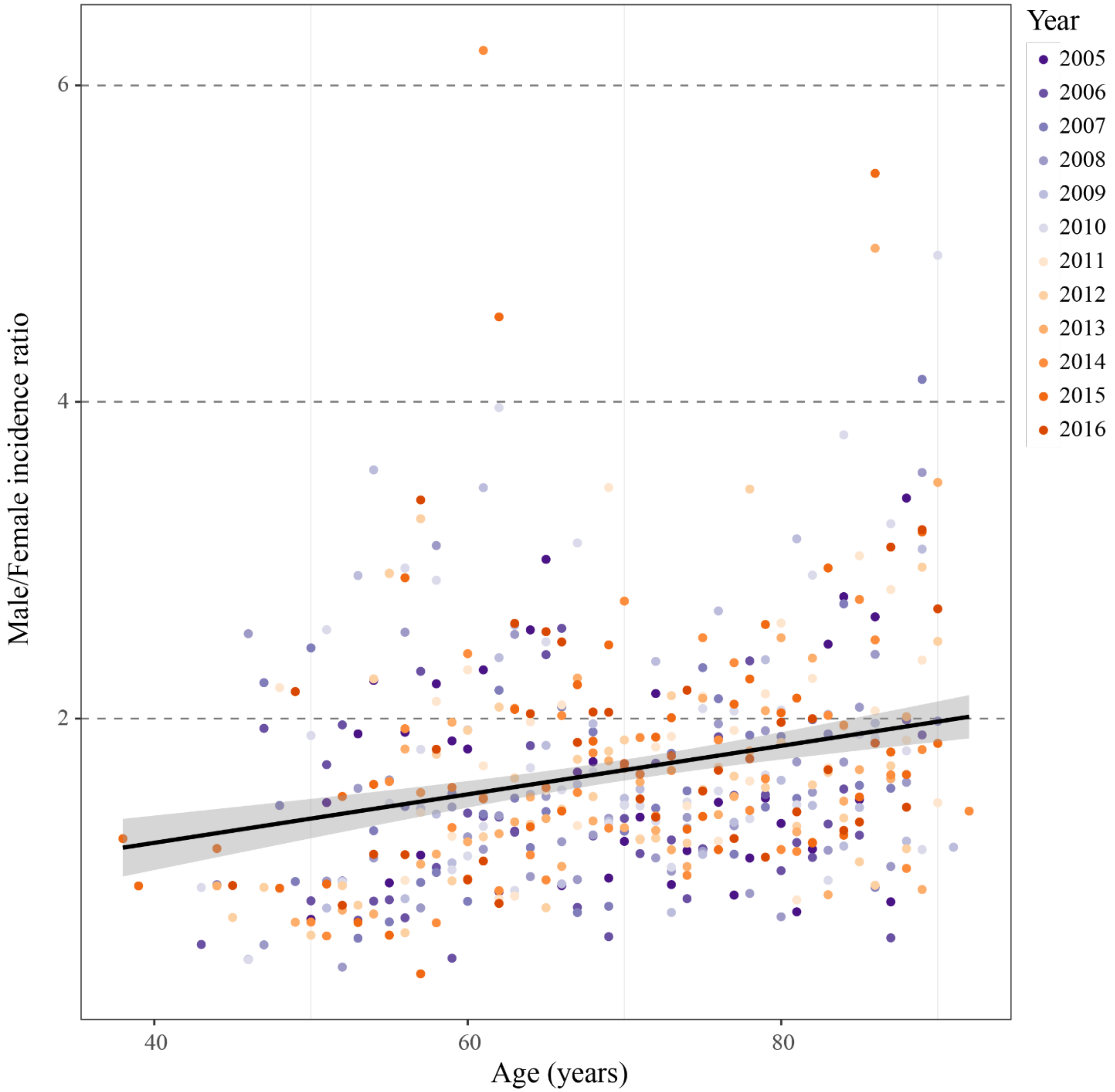

Supplementary Figure 2. Increase in the male/female incidence ratio with age.
Incidence was calculated for 100,000 person-years by dividing the number of new PD cases in each year by the size of the corresponding age- sex-matched population in the same year (normalized to 100,000). Each point represents the male/female incidence ratio for a particular year in a particular age. The straight line indicates the linear fit (and 95% confidence interval) of the data. Only data points for each year which the number of new PD cases for both sexes > 2 are shown. Linear regression of the data, adjusted for the observation year, indicates significant effect of age, with 1.5% increase in incidence ratio for every year of life ($\beta = 0.015$, $p < 5.2^{-8}$). Similar effect was observed based on Poisson regression analysis.

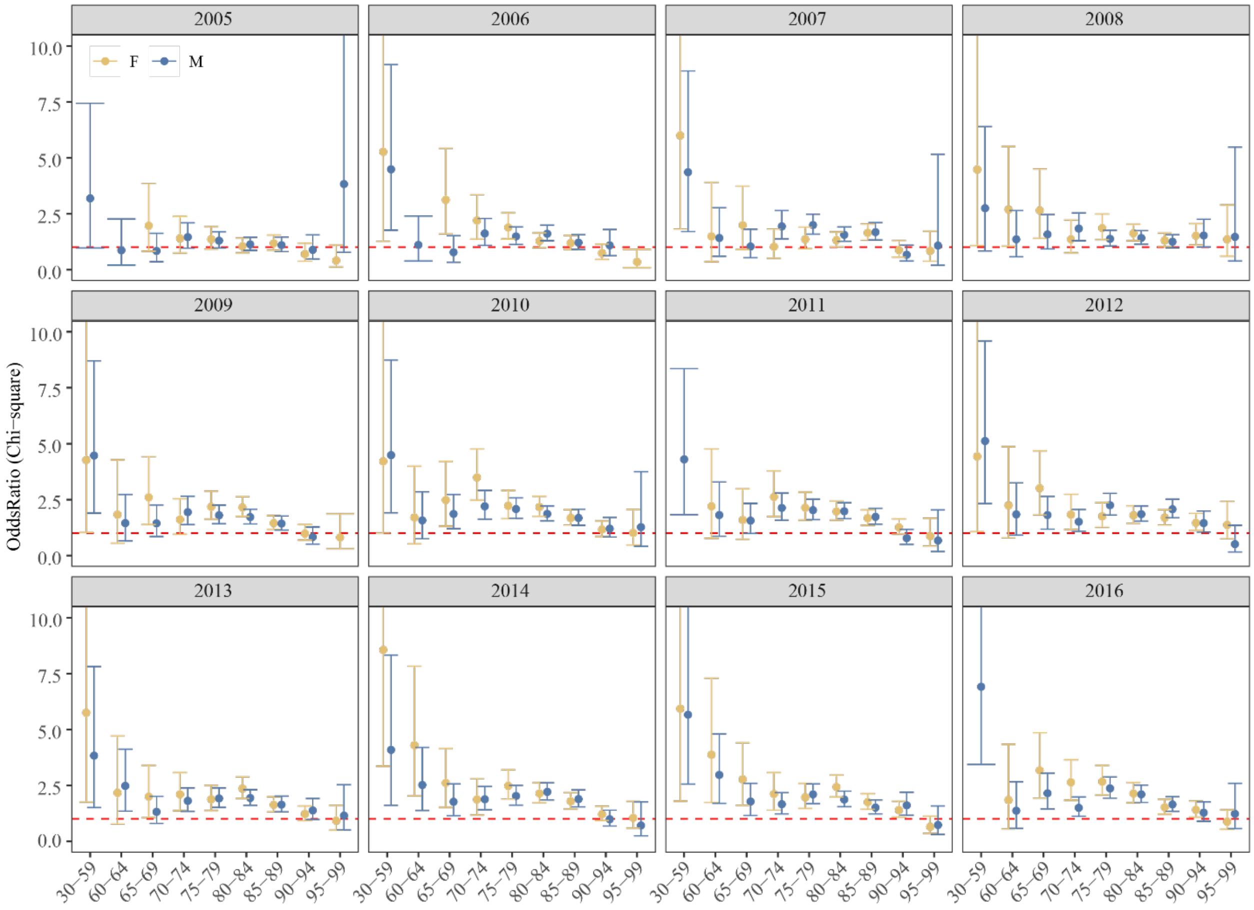

Supplementary Figure 3. Yearly death odds ratios for individuals with PD compared to general population

Estimated death odds ratios (individuals with PD compared to controls) and 95% confidence intervals calculated for each year of observation.

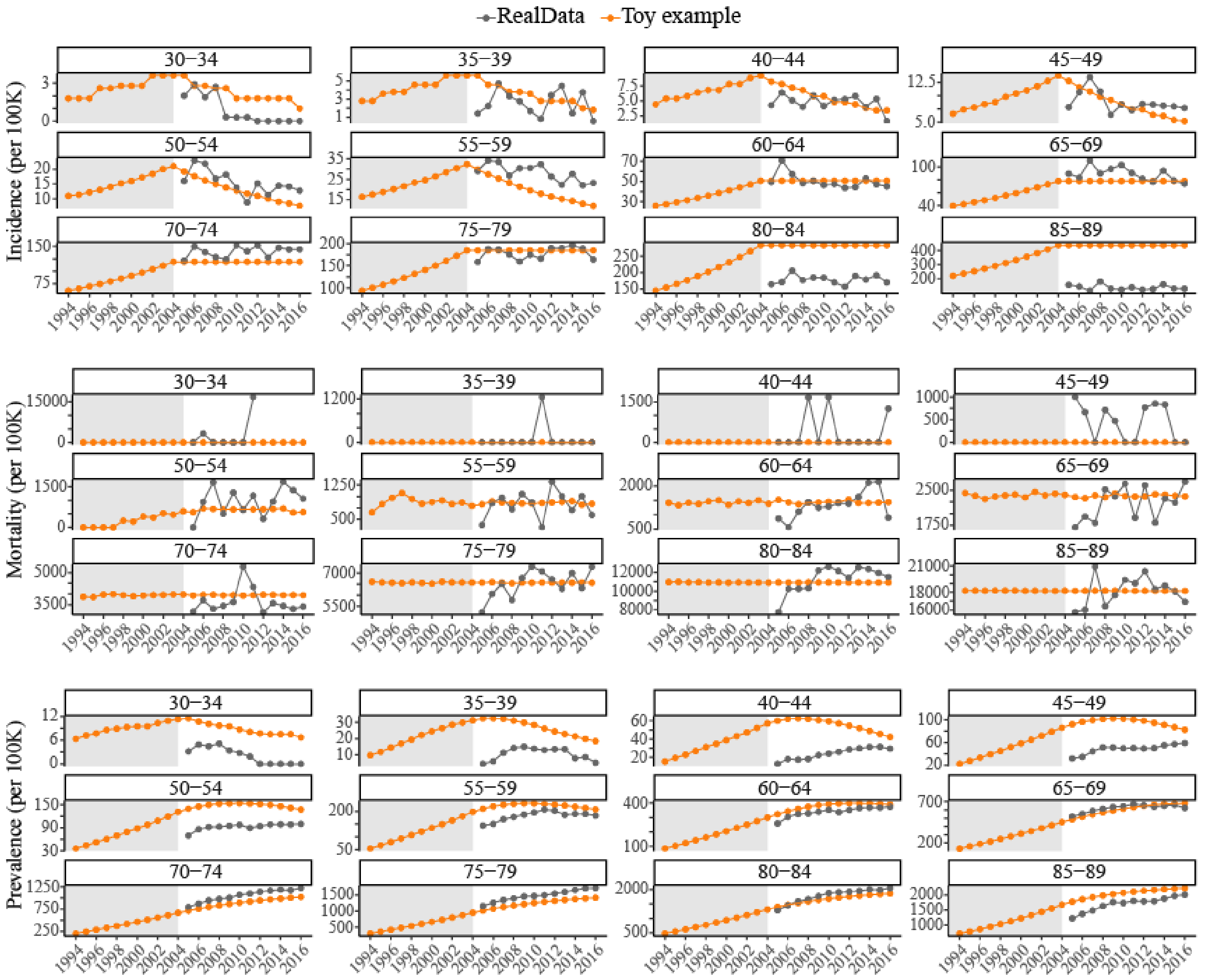

Supplementary Figure 4. Toy example demonstrating an increase in prevalence due to temporal increase in incidence taking place prior to the observation period.

To illustrate how increase in PD prevalence could occur without concordant change in incidence/mortality during our observation period (2004-2016), we simulated a hypothetical scenario in which an increase in incidence in all age groups happened prior to the observation period. For the purpose of the simulation, age-specific population size was assumed to be constant throughout the simulation period, and equal across all age groups. For simplicity, this number was chosen as 100,000. Mortality was kept constant throughout the years, and the age-specific values were chosen to be representative of the values in our data. After seeding the incidence values in 1994 for ages 30-89, the incidence of all ages was set to yearly increase by 7% for 10 years (1994-2004, "pre-observation period"). To match with the real data, incidence among ages < 60 was set to decrease by 8% every year and remain constant for ages >= 60 over the following years (2004-2016, "observation period"). The age-specific prevalence in 1994 was set as 3.5*age-specific incidence. Prevalence in each of the following years was then propagated for each age and year as following:

$$PrevalenceRaw_{Age,Year} = PrevalenceRaw_{Age-1,Year-1} + PDnew_{Age,Year} - PDdeath_{Age-1,Year-1}$$

when $PDdeath_{Age-1,Year-1} = round(Mortality_{Age-1} * \mathrm{PrevalenceRaw}_{Age-1,Year-1})$.

As a final step, we recalculated the mortality to account for the rounding of PDdeath. Shown are the mean simulated values in each age group (orange). The real, observed data is shown in dark grey. The code for the simulation and plotting is accessible through the repository https://github.com/ltoker/PDepidemiology. The individual data points are available through the same repository.
